# A Dangerous Couple: Sequential Effect of Phosphorus Flame-Retardant and Polyurethane Decrease Locomotor Activity in Planarian *Girardia tigrina*

**DOI:** 10.3390/biology13050337

**Published:** 2024-05-12

**Authors:** Dora Bjedov, Rone S. Barbosa, Danielle Palma de Oliveira, Daniel Junqueira Dorta, Maíra Ignacio Sarmento, Renato Almeida Sarmento, Ana L. Patrício Silva, Carlos Gravato

**Affiliations:** 1 Centre for Ecology, Evolution and Environmental Changes (cE3c) & CHANGE—Global Change and Sustainability Institute, Faculdade de Ciências, Universidade de Lisboa, Campo Grande, 1749-016 Lisbon, Portugal; dora.bjedov@gmail.com; 2 Departamento de Biologia Animal, Faculdade de Ciências, Universidade de Lisboa, Campo Grande, 1749-016 Lisbon, Portugal; 3 Graduate Program in Forestry and Environmental Sciences, Universidade Federal do Tocantins, Campus de Gurupi, Gurupi 77402-970, TO, Brazilmairaig@uft.edu.br (M.I.S.); rsarmento@uft.edu.br (R.A.S.); 4 School of Pharmaceutical Sciences, University of São Paulo, Campus de Ribeirão Preto, Ribeirão Preto 77402-970, SP, Brazil; dpalma@usp.br; 5 National Institute of Science and Technology for Detection, Toxicological Evaluation and Removal of Micropollutants and Radioactive Substances (INCT-DATREM), Araraquara 14800-060, SP, Brazil; djdorta@ffclrp.usp.br; 6 Department of Chemistry, Faculty of Philosophy, Science and Letters at Ribeirão Preto, University of São Paulo, Ribeirão Preto 14040-901, SP, Brazil; 7 Centre for Environmental and Marine Studies (CESAM), Departament of Biology, University of Aveiro, Campus Universitário de Santiago, 3810-193 Aveiro, Portugal; ana.luisa.silva@ua.pt

**Keywords:** aluminium diethylphosphinate, microplastics, organic pollutants, behavioural endpoints, molecular biomarkers

## Abstract

**Simple Summary:**

Exploring the interactions between organophosphorus flame retardants (OPFRs), microplastics, and freshwater organisms is essential for comprehending the dynamics within freshwater ecosystems to anticipate the potential impacts of organic pollutants and plastic particles. To address this need, the current study examined the exposure effects of 10 mg L^−1^ of flame-retardant aluminium diethylphosphinate (ALPI), 10 μg mg^−1^_liver_ of microplastics polyurethane (PU), and their combination on the freshwater planarian *Girardia tigrina*. Twenty-four-hour exposure of *G. tigrina* to both ALPI and PU resulted in a sequential effect reflected in a significant reduction in locomotor activity, i.e., exposure may involve a combination of direct neurotoxic effects and the indirect energetic costs associated with the adaptive responses to mitigate the adverse effects. Other biochemical responses, e.g., oxidative stress and metabolic responses, remained unaffected compared to control. Incorporating behavioural indicators into toxicological assays enhances the predictive power of these assessments, enabling a more accurate evaluation of the ecological consequences of pollutant exposure. This integrated approach not only improves our understanding of the complex interactions between organisms and their environment, but also informs more effective strategies for mitigating the detrimental effects of mixtures of pollutants on freshwater ecosystems.

**Abstract:**

Understanding the interplay among organophosphorus flame retardants (OPFRs), microplastics, and freshwater organisms is crucial for unravelling the dynamics within freshwater environments and foreseeing the potential impacts of organic pollutants and plastic contamination. For that purpose, the present research assessed the exposure impact of 10 mg L^−1^ flame-retardant aluminium diethylphosphinate (ALPI), 10 μg mg^−1^_liver_ microplastics polyurethane (PU), and the combination of ALPI and PU on the freshwater planarian *Girardia tigrina*. The exposure to both ALPI and PU revealed a sequential effect, i.e., a decrease in locomotor activity, while oxidative stress biomarkers (total glutathione, catalase, glutathione S-transferase, lipid peroxidation) and metabolic responses (cholinesterase activity, electron transport system, and lactate dehydrogenase) remained unaffected. Despite this fact, it was possible to observe that the range of physiological responses in exposed organisms varied, in particular in the cases of the electron transport system, cholinesterase activity, glutathione S-transferase, catalase, and levels of total glutathione and proteins, showing that the energetic costs for detoxification and antioxidant capacity might be causing a lesser amount of energy allocated for the planarian activity. By examining the physiological, behavioural, and ecological responses of planarians to these pollutants, insights can be gained into broader ecosystem-level effects and inform strategies for mitigating environmental risks associated with OPFRs and microplastic pollution in freshwater environments.

## 1. Introduction

In recent years, the proliferation of anthropogenic chemicals and materials has brought about growing concerns regarding their impact on the environment and living organisms [1]. Among these concerns are the widespread use of organophosphorus flame retardants (OPFRs) and the ubiquitous presence of microplastics in aquatic ecosystems [2]. While OPFRs and microplastics can each independently induce adverse effects on biota, their combined presence presents substantial ecological risks. Furthermore, most commercially available microplastic (polyurethane) may contain OPFR as additives. Such additives, however, in polyurethane or other polymers designed for thermal stability and heat resistance, might not be chemically bonded to these polymers, and may leach to the surrounding environment [3]. OPFRs have been detected in freshwaters and wastewaters [4]; therefore, comprehending their interactions with freshwater organisms, such as planaria, is essential for evaluating their environmental impacts [1,2]. OPFRs are a class of chemicals commonly added to a variety of consumer products and materials to reduce their flammability that can be found in furniture, building materials, textiles, floor polishes, coatings, epoxy resins and engineering thermoplastics, and, as in [5], polyurethane plastics used as insulation foams and various products related to furniture, building and electronics [6]. While effective at preventing fires, OPFRs are often not chemically bound to these products and materials and can gradually leach out, being detected in various environmental compartments, particularly in water bodies, where they can accumulate and persist due to their chemical properties. Concerns over the potential adverse effects of OPFRs on aquatic organisms have prompted extensive research into their environmental fate, toxicity, and ecological impacts [5,7]. Similarly, microplastics, plastic particles measuring less than five mm in size, have emerged as a global environmental concern [8]. These particles originate from the breakdown of larger plastic items or are intentionally manufactured for use in products such as personal care items, e.g., exfoliating scrubs, cosmetics, hair care products, and industrial abrasives, e.g., sandblasting materials, polishing compounds, and composite materials [9,10]. Microplastics are pervasive in freshwater environments worldwide, posing risks to aquatic organisms through ingestion, physical harm, and the release of associated chemical additives and contaminants [8].

Understanding the interactions between OPFRs, microplastics, and freshwater biota is essential for elucidating the complex dynamics within freshwater ecosystems and predicting the potential long-term consequences of chemical and plastic pollution [11]. The impact of microplastics on the availability of environmental pollutants should not be underestimated, given that their influence has been shown to be significant. It is crucial to analyse diverse scenarios to extract precise insights into the ecotoxicological implications of the “Trojan horse” effect of microplastics, i.e., microplastics can act as a vector for the uptake of pollutants to aquatic organisms [12]. Additionally, it is important to consider that the presence of microplastics may also heighten the availability of emerging pollutants such as flame-retardant compounds ([13] and references therein).

Freshwater planaria serve as valuable indicators of environmental quality and are frequently used in ecotoxicology studies to assess the effects of contaminants on aquatic organisms [14]. Employing freshwater planarians in toxicological research shows considerable potential. These organisms are frequently utilised to investigate the toxic effects of xenobiotic compounds, particularly those stemming from human activities. Additionally, freshwater planarians have been proposed as bioindicators of freshwater environment quality as is reflected in their sensitivity to environmental stressors and relatively simple biology [15]. Due to their adaptability to laboratory culturing conditions, freshwater planarians are highly suitable non-target organisms for investigating sub-lethal effects of contaminants across a broad spectrum of endpoints. These may include regeneration, locomotion, feeding, bioaccumulation, and biochemical parameters, among others [16]. Since planarians are small-sized predators commonly found in many freshwater systems [17], they can be excellent model organisms to be used in ecotoxicological research. Planarians are highly sensitive to changes in their environment, including exposure to various chemical pollutants and toxins [15]. They can serve as reliable indicators of environmental contamination because their physiological responses to toxic substances can be readily observed and measured. Planarians exhibit various behaviours that can be affected by exposure to toxic pollutants, such as changes in locomotion, feeding, and response to stimuli. These behavioural changes can serve as sensitive indicators of sublethal toxicity [15,16]. Therefore, planarians are ideal model organisms to assess the direct and indirect effects of pollutants, e.g., OPFR and/or microplastics, along trophic chains; thus, they hold great potential as bioindicators of the ecological integrity of freshwaters. Also, freshwater planarians are useful model species in behavioural and biomedical studies as they are easily maintained in the laboratory and display specific behavioural responses to psychoactive substances. Moreover, planarians are relatively easy to handle and maintain in laboratory conditions. They can be cultured in controlled environments and their behaviours, growth rates, and reproductive cycles can be closely monitored over time [15,18]. Planarians are well-characterised animal models in neurobiology research and their nervous system shares features with vertebrates in terms of cell morphology and physiology [19].

Research conducted on planarians has documented the uptake of polyethylene microspheres and polyethylene terephthalate microfibers. These microplastics were found in concentrations ranging from 12 to 60 μg mg^−1^ in contaminated food sources, particularly liver, without observable alterations in their feeding behaviour, food consumption, or regenerative capabilities [20]. However, this exposure led to a significant reduction in the thickness of the gut epithelium and the lipid content of enterocytes, accompanied by apoptotic cell death induction and a decrease in growth rate [20]. The exposure of planarians to polystyrene microspheres at concentrations of 10, 50, and 100 μg mg^−1^ in liver tissue resulted in a notable decrease in both body and blastema, i.e., the regenerative area with fast proliferation of tissue due to differentiation of neoblasts triggered by physical injury. This decline indicated a delay in growth and regeneration processes [21]. Concurrently, the proliferation and differentiation of stem cells were hindered, and there was a decrease in the proportion of mitotic stem cells [21]. Additionally, polystyrene microspheres appeared to induce oxidative stress in planarians, as evidenced by significant alterations in the activity of antioxidant enzymes, e.g., glutathione S-transferase and catalase [22].

In the present research, we assessed the 24 h exposure impact of organophosphorus flame-retardant aluminium diethylphosphinate, polyurethane microplastic particles, and their combination on the freshwater planarian *Girardia tigrina*. The primary aim of our investigation was to examine the repercussions of individual and combined acute exposure to aluminium diethylphosphinate as well as polyurethane microplastic particles ingested by freshwater planarians (*G. tigrina*) through the consumption of bovine liver. Morphological, behavioural, metabolic, and biochemical endpoints at sub-lethal levels were determined. In particular, we evaluated the weight, length, and locomotor activity of planarians as biomechanical parameters, as well as the effects on detoxification (glutathione S-transferase), antioxidant capacity (level of total glutathione and activity of catalase), oxidative stress (level of lipid peroxidation), aerobic (electron transport system activity) and anaerobic (lactate dehydrogenase) consumption of energy, and levels of protein and acetylcholinesterase activity as a proxy of activity and neurotoxicity biomarker.

## 2. Materials and Methods

### 2.1. Chemicals

Spherical aliphatic polyurethane (PU) microplastics are characterised by their unmodified and non-stained state with fluorescent dyes, presenting as a white powder with no discernible odour and exhibiting a density of 1.05 g cc^−1^ at 25 °C. These PU microplastic particles have an average size ranging between 7 and 9 μm, with a maximum size of 31 μm. Aluminium diethylphosphinate (ALPI, CAS number 225789–38–8, MW 390.27) was donated by Clariant (trade name of Exolit^®^ OP 1230) (Clariant, Germany). ALPI (10 mg L^−1^) was prepared in commercial natural water (Table 1). PU microplastics, in certain forms or formulation, are naturally quite inflammable since they are one of the polymer types that heavily rely on the addition of flame retardants. ALPI, on the other hand, is a flame retardant used in specific forms and formulations of PU.

### 2.2. Test Organism

The planarians, *G. tigrina*, were maintained at the Faculty of Sciences, University of Lisbon (Lisbon, Portugal), since 2019. Planarians were kept under controlled conditions using commercial natural water (pH 6.01 ± 1) with specific composition parameters to ensure that the planarians have access to essential minerals and nutrients as well as stable pH conditions necessary for their growth, development, and overall homeostasis (Table 1). The water was aerated and maintained at 22 ± 1 °C in a dark environment. Seeing as exposure to prolonged light can disrupt their natural behaviours, including feeding and movement patterns, the planarians were kept in a dark environment to mimic their natural habitat conditions and to minimise stress. Planarians were fed with bovine liver ad libitum twice a week. Organisms ranging from 10 to 14 mm in length were selected for all the experimental procedures after a fasting period of seven days. The planarians used (*n* = 40 in total) were in good condition without lesions and changes in their locomotor behaviour in response to light.

### 2.3. Exposure to Phosphorous Flame Retardant and Polyurethane

Planarians were divided into four glass recipients (*n* = 10 per recipient) containing 40 mL of commercial natural water previously detailed. Two groups of 10 planarians were fed with 10 mg of homogenised bovine liver for 5 h in dark conditions at 22.00 ± 1 °C. The other two groups were fed with 10 mg homogenised bovine liver containing 10 µg mg^−1^_liver_ of polyurethane (PU) particles in the size range of 3–5 μm during 5 h in dark conditions at 22.00 ± 1 °C. After the feeding period, the remaining liver was removed. PU particles used in this study remain unchanged and unstained. The size range of the plastic particles was selected based on the size of the particles ingested by planarians in previous research works [20,21].

One of the two groups fed with liver only were exposed during 24 h to water only, i.e., the control group, and the second group was exposed to 10 mg L^−1^ organophosphorus flame-retardant aluminium diethylphosphinate (ALPI) for the same period (referred to as ALPI treatment [23]). Moreover, two groups of planarians fed with liver containing PU particles were exposed, during 24 h, to water only to assess the effects of the PU particles present in the diet (referred to as PU treatment [24]) and to 10 mg L^−1^ ALPI (referred to as ALPI + PU treatment) to assess the effects of ALPI in planarians fed with PU particles. After the feeding period, the remaining liver was removed and the water was completely renewed.

### 2.4. Biomechanical Response

Following the feeding period (5 h) and the exposure period (24 h), the locomotor velocity, body mass, and body weight of planarians of each group were individually assessed. Changes in locomotor velocity, body mass, and weight can reflect physiological responses, metabolic changes, or nutritional effects due to toxic exposure. The locomotor activity was determined in a separate glass container with a diameter of 55 cm. This container was positioned on a sheet of millimetre paper with grid lines spaced accurately to 0.5 cm. A small volume of natural commercial water was carefully regulated to facilitate the smooth locomotion of the planarians while facilitating accurate observation and recording of their movements. Planarians were individually positioned in the centre of the container and distance was recorded in video over a 3 min period. The number of grid lines crossed and re-crossed by each planarian was observed on video, allowing for the determination of the locomotor velocity (crossed gridlines per min) for each individual. Subsequently, 10 planarians of each group were individually placed in 2 mL microtubes and stored at −80 °C for sample preparation. Variability in locomotor velocity measurements was controlled using consistent observation periods and tracking methods as described, and variability in body mass and weight measurements was minimised using precision scales and standardised procedures for weighing planarians.

### 2.5. Sample Preparation

Each sample was prepared by placing one planarian into a microtube along with 8 small spheres. Ultra-pure water (1000 μL) was added to each microtube. The samples were then homogenised using a Tissue Lyser set to 30 Hz for 2 + 2 min. Subsequent to homogenisation, 1000 μL of ultra-pure water was added for the final volume of 2000 μL. Samples were centrifuged at 1000× *g* for 5 min at 4 °C. Pellet was discarded, and the supernatant was divided into aliquots. In the aliquot designated for lipid peroxidation analysis, BHT (2,6-Di-tert-butyl-4-methylphenol (BHT) diluted with 4% methanol was added into a sample. For the aliquots intended for electron transport system determination, a solution comprising Tris-(hydroxymethyl)-aminomethane (Tris base), polyvinylpyrrolidone (PVP), MgSO_4_, and Triton X-100 was added. Following aliquotation and homogenisation, the samples were promptly stored at −80 °C until biomarker analysis.

### 2.6. Oxidative Stress

Homogenate samples, as well as blank controls, were measured in four technical replicas, and all methods were adjusted for a microplate reader. Glutathione S-transferase (GST) activity was performed based on the Habig et al. [25] method. The reaction mixture contained a sample, potassium phosphate buffer, reduced glutathione (GSH), and 1-cloro-2,4-dinitrobenzeno (CDNB). Absorbance was measured at a wavelength of 340 nm every 20 s for 5 min. Specific activity was calculated based on the molar extinction coefficient (ε) 9.6 × 10^3^ M^−1^ cm^−1^. Catalase (CAT) activity was performed according to Claiborne [26]. A decrease in absorbance was measured in a mixture of sample, potassium phosphate buffer, and hydrogen peroxide (H_2_O_2_) at 240 nm for 2 min. Specific activity was calculated based on ε = 40 M^−1^ cm^−1^. Total glutathione (TG) levels in planarian samples were determined with potassium phosphate buffer, β-nicotinamide adenine dinucleotide 2′-phosphate reduced tetrasodium salt (β-NADPH), 5,5′–dithiobis–(2–nitrobenzoic acid) (DTNB), and glutathione reductase (GR). Absorbance was measured at 412 nm for 3 min. The concentration of TG was calculated based on a standard curve using GSH. Lipid peroxidation (LPO) concentrations were assessed by measuring thiobarbituric acid reactive substances (TBARS) following the protocols established by Ohkawa et al. [27] and described in detail in Silva et al. [28]. The reaction mixture contained sample, trichloroacetic acid (TCA), 2-thiobarbituric acid (TBA), trizma hydrochloride, Tris—HCl, and diethylene triamine pentaacetic acid (DTPA). The mixture was incubated at 100 °C for 60 min and subsequently centrifuged at 9000 rcf for 5 min at 25 °C. Absorbance was read at 535 nm, and ε = 1.56 × 10^5^ M^−1^ cm^−1^ was used to calculate the LPO levels. The selection of the aforementioned specific biomarkers reflects a strategic approach to assess the potential adverse effects by increasing metabolism that leads to oxidative stress in planarians if the detoxification and antioxidant capacities do not cope with increased reactive oxygen species produced.

### 2.7. Metabolic Biomarkers

Cholinesterase (ChE) activity was determined based on the solution containing potassium phosphate buffer, acetylthiocholine iodide, and DTNB. Absorbance was measured at 414 nm, every 20 s for 5 min. Specific activity was calculated based on ε = 13.60 × 10^3^ M^−1^ cm^−1^. Electron transport system (ETS) activity was measured according to the protocol described in [28]. The following reagents were used: β-NADPH, reduced β-nicotinamide adenine dinucleotide (β-NADH), and *p*-iodonitrotetrazolium (INT). The absorbance was monitored at 490 nm for 3 min. Activity was calculated based on ε = 1.59 × 10^4^ M^−1^ cm^−1^. Lactate dehydrogenase (LDH) activity was analysed using Tris, NaCl, NADH, and pyruvic acid. Absorbance was measured at 340 nm every 20 s for 5 min. Activity was calculated based on ε = 6.30 mM^−1^ cm^−1^. The total protein content was determined using Bradford’s method which was adapted for microplate analysis. Bovine serum albumin was utilised to establish the calibration curve, with absorbance readings taken at 592 nm. The selection of specific biomarkers reflects a strategic approach to assess the potential disruption of physiological responses in planarians such as energy production and neurotransmission.

### 2.8. Data Analysis

The parameters of locomotor activity, body mass, body weight, GST, CAT, TG, LPO, ChE, ETS, LDH, and protein content underwent analyses to evaluate the normality of distribution. The normality of the data was visually assessed with QQ plots and histograms, followed by the Shapiro–Wilk test. Confirming the assumed normality of the data, parametric tests were used. A one-way analysis of variance (ANOVA) was performed to investigate the differences between the parameter response compared to the control group. Multiple comparisons, i.e., comparing the mean of each column (response variable) to the mean of the control group, were determined by Dunnett’s post hoc test. Statistical significance was considered at a *p*-value ≤ 0.05. Results are presented as barplots, with mean ± standard deviation (SD). Statistical analyses and data visualisations were performed using GraphPad Prism software version 7.0 for Windows (GraphPad Software, La Jolla, CA, USA).

## 3. Results and Discussion

**Figure 1:**
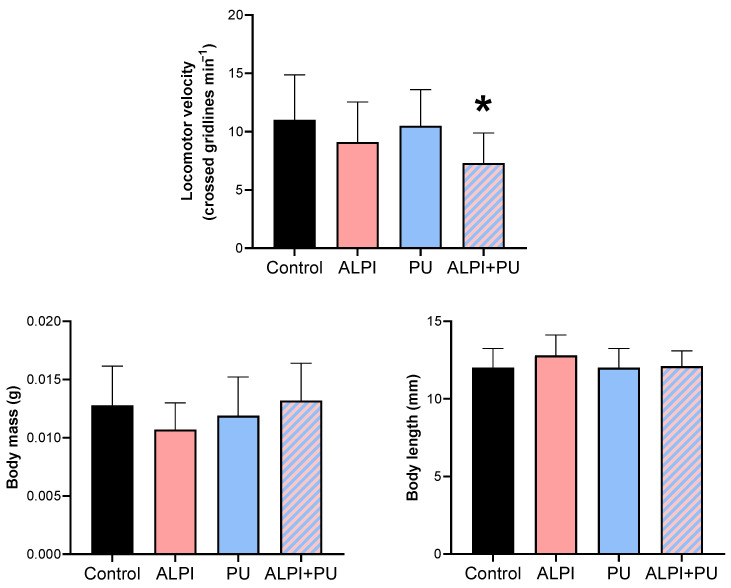
Biomechanical response—locomotor velocity (crossed gridlines min^−1^), body mass (g), and body length (mm) in planarians *G. tigrina* exposed to 10 mg L^−1^ flame-retardant aluminium diethylphosphinate (ALPI), 10 μg PU mg^−1^_liver_ plastic polymer polyurethane (PU), and the combination of ALPI and PU. Results are presented as mean ± standard deviation, SD. Statistical differences between the exposures compared to the control (ANOVA, followed by Dunnett’s multiple comparisons test) are presented with * (*p* < 0.05).

The locomotor activity of planarians was the only biomechanical response that was significantly altered in organisms exposed to PU particles and ALPI (Table 2, Figure 1) through the diet and water, respectively. A significant difference was observed only in locomotor velocity post exposure to both ALPI and PU particles compared to the control group (*p* < 0.05). Changes in post-exposure planarian behaviour, i.e., locomotion, appeared to be useful endpoints that can detect sequential sub-lethal effects of ALPI and PUs. The observed difference in locomotor velocity may be due to the combined neurotoxic effects of ALPI and PU particles. Interestingly, exposure to PU particles or ALPI individually did not provoke a significant response in locomotor activity. The exposure to concentration/dose of ALPI or PU particles was not potent enough to elicit a response; these components may interact in a way that enhances their effects when combined, leading to a significant response (Table 2, Figure 1). Additionally, ALPI and PU particles have a sub-threshold effect, and it appears that the exposure concentration did not reach the level required to trigger a response in planarian locomotor activity. On the other hand, ALPI and PU particles may affect different neurological pathways or neurotransmitter systems involved in locomotor activity. It is likely that simultaneous exposure to both ALPI and PU particles induces an interaction at a neurological level to produce a significant response (Table 2, Figure 1). Previous research showed the neurotoxic effects of organophosphorus flame retardants on freshwater planarians [29]. However, the locomotor activity of planarians was not significantly altered when exposed to a single dose of ALPI which indicate that the concentration, exposure period or the mode of exposure may not be sufficient to induce a response (Table 2, Figure 1). The impaired locomotor function may stem from disruptions in neurotransmission pathways and alterations in the activity of various biomarkers. The flame-retardant DE-71 adversely affected the cholinergic system and locomotor activity in *Danio rerio* larvae by perturbing calcium homeostasis [30]. That being said, planarian locomotion is regulated by the dopaminergic system [31] and it may be affected by the combination of ALPI and PU. As a result, impaired locomotor behaviour, marked by decreased mobility, can pose a notable ecological risk and potentially endanger survival [32]. Post-exposure alterations in locomotor activity have also been utilised as indicators of stress induced by ammonia in the planarian *Polycelis felina* [33]. The potential impact of PU particles and microplastic particles in general on the locomotion of planarians has been previously reported, and results in this study correspond with the absence of PU effect on locomotor activity shown in [28]. However, alterations in locomotor activity have been documented in organisms exposed to microplastics or prey contaminated with microplastics. The changes have been observed in invertebrates including *Crassostrea gigas*, *Daphnia magna*, and *Artemia franciscana* as well as vertebrates such as *Danio rerio* larvae [34,35,36,37,38]. The overall decrease was evident in reductions in average speed and distance moved. Changes in the locomotor activity of predators could further result in negative impacts on their hunting behaviour, exploration competence, and ability to escape from other predators, thereby reducing their fitness and potentially disrupting ecosystem function. Behavioural changes arise from a biochemical and physiological biomarker approach, potentially linked to modifications at higher levels of biological organisation [33,39]. Behaviour has been proposed as a sensitive indicator of ecotoxicological impact, as it often responds earlier than traditional endpoints such as growth in terms of body mass and length, as well as reproduction [33]. The observation of decreased locomotor activity in planarians emphasises the value of behavioural parameters as supplementary tools in evaluating the sub-lethal effects of various pollutants [40]. The present research provides additional support for the suitability of planarians in ecotoxicity assessment. Behavioural endpoints serve as valuable instruments for evaluating the external manifestation of neurotoxicity induced by environmental pollutants, representing a comprehensive, organism-wide response that bridges biochemical and physiological processes [39,41].

**Figure 2:**
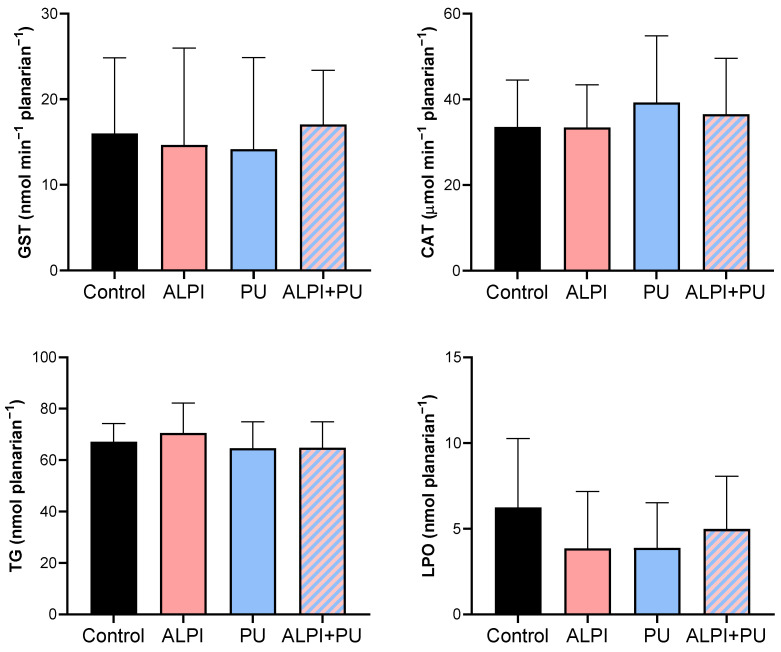
Oxidative stress biomarkers—glutathione S-transferase (GST, nmol min^−1^ planarian^−1^), catalase (CAT; µmol min^−1^ planarian^−1^), total glutathione (TG, nmol planarian^−1^), and lipid peroxidation (LPO, nmol planarian^−1^) in planarians, *G. tigrina* exposed to 10 mg L^−1^ flame-retardant aluminium diethylphosphinate (ALPI), 10 μg PU mg^−1^_liver_ plastic polymer polyurethane (PU), and the combination of ALPI and PU. Results are presented as mean ± standard deviation, SD.

Given the potential of flame retardants and microplastic to induce oxidative stress, we conducted a biochemical analysis to assess the status of the defence system in exposed planarians, *G. tigrina* (Table 3, Figure 2). The antioxidant defence system comprises both enzymatic and non-enzymatic antioxidants, which function by neutralising reactive oxygen species (ROS) and protecting the planarians against the increase in oxidative stress. The initial defence line against oxidative stress includes the antioxidant enzymes, CAT, and glutathione-dependent enzymes. CAT breaks down H_2_O_2_ into water and molecular oxygen [42] and GST plays a pivotal role in pollutant metabolism by facilitating the conjugation of GSH with electrophilic metabolites, thus aiding in their excretion [43]. Furthermore, an imbalance between the production of ROS and the antioxidant defence mechanisms may lead to oxidative damage to cellular components, including lipids. This process, known as lipid peroxidation, results in the formation of lipid peroxides and other reactive by-products, ultimately compromising membrane integrity and cellular function. Planarians exposed to ALPI, PU and the combination of ALPI and PU did not exhibit any significant changes in enzymatic activity (GST, CAT) or concentration of TG and LPO (*p* > 0.05, Table 3, Figure 2). This may be due to the exposure concentrations and duration of the acute (24 h) exposure. The concentration of 3 and 30 µg L^−1^ BDE-47 and the concentration of 30 mg L^−1^ ALPI caused changes in CAT activity, but not in GST activity, as shown in *D. rerio* [44,45]. Exposure to polyvinyl microplastics led to alterations in haematology, antioxidant enzyme levels, LPO, and acetylcholinesterase activity in the African catfish *Clarias gariepinus* [46,47]. However, this is not the case in the present study. Toxic effects often depend on the dose and duration of exposure, and if these are below a threshold level [48], observable changes may not occur. Planarians might have exhibited adaptive responses to the exposure, upregulating their antioxidant defence systems in response to oxidative stress induced by PU, ALPI, and/or their combination. Despite the exposure to the pollutants, this upregulation could have maintained CAT and GST activities, TG levels, and LPO within the ranges of control. Therefore, interactions between PU and ALPI, either synergistic or antagonistic, could have affected the observed results in a way that the combination of PU and ALPI may have resulted in counteractive effects on oxidative stress pathways, leading to no change in CAT and GST activity as well as TG and LPO levels.

**Figure 3:**
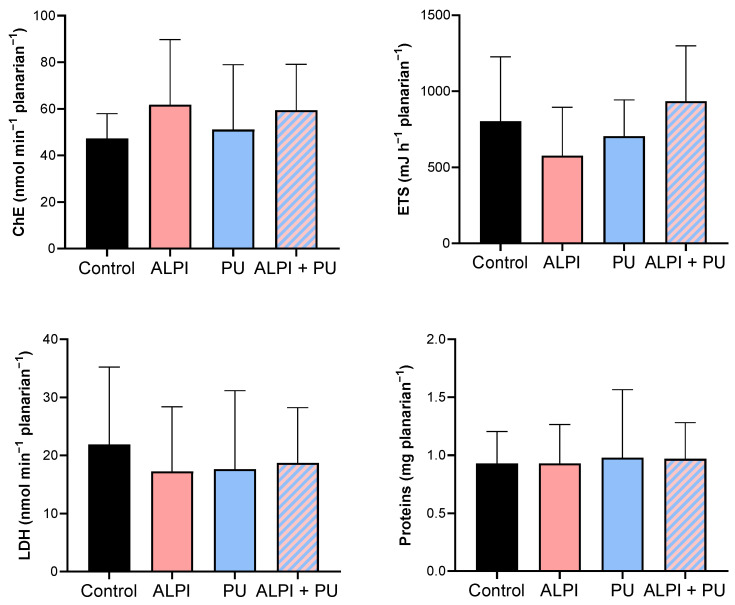
Metabolic biomarkers—cholinesterase activity (ChE, nmol min^−1^ planarian^−1^), electron transport system (ETS, mJ h^−1^ planarian^−1^), lactate dehydrogenase activity (LDH, nmol min^−1^ planarian^−1^) and protein content (mg planarian^−1^) in planarians, *G. tigrina* exposed to 10 mg L^−1^ flame-retardant aluminium diethylphosphinate (ALPI), 10 μg PU mg^−1^_liver_ plastic polymer polyurethane (PU), and the combination of ALPI and PU. Results are presented as mean ± standard deviation, SD.

In the present study, none of the examined pollutants—ALPI, PU, and a combination of ALPI and PU—showed significant alterations in the activity of ChE in exposed planarians (*p* > 0.05, Table 4, Figure 3). In contrast, research on various polybrominated diphenyl ethers (PBDEs) suggests their potential to impact the nervous system via the acetylcholine system. For instance, the PBDE mixture DE-71 notably suppressed AChE activity in *D. rerio* [30,49]. Similarly, exposure to BDE-209 and its combinations with BDE-47 and BDE-99 significantly inhibited AChE activity in *Carassius auratus* [50]. The electron transport system (ETS) did not show any significant differences when exposed to ALPI, PU, or the combination of ALPI and PU compared to control (*p* > 0.05, Table 4, Figure 3). This finding could imply that the toxic effects of ALPI, PU, and their combination may be exerted through mechanisms other than direct interference with cellular respiration. Alternatively, it may suggest that planarians possess mechanisms to maintain the integrity of their electron transport system even in the presence of these pollutants. ETS has been evaluated in planarians, suggesting that energy allocation refers to the prioritisation of energy resources within a planarian to support homeostasis. Changes in energy allocation patterns can occur in response to environmental stressors or challenges, and these changes may have implications for overall health and fitness [28,51,52]. LDH facilitates the conversion of pyruvate to lactate within the anaerobic pathway of cellular energy generation [53]. Research has demonstrated that exposure to environmental pollutants can alter LDH activity [54]; however, that is not the case in the present study. We detected no significant changes in LDH activity following exposure to ALPI, PU, and the combination of ALPI and PU compared to the control group (*p* > 0.05; Table 4, Figure 3). Interestingly, in research by Nematdoost Haghi and Banaee [55], microplastics alone did not change LDH activity in *Cyprinus carpio*, but when combined with the herbicide paraquat, an increase in LDH activity was detected. Protein content remained unaffected in planarians after exposure to ALPI, PU, and the combination of ALPI and PU, suggesting these pollutants did not significantly alter overall protein synthesis or degradation processes (*p* > 0.05, Table 4, Figure 3). Changes in protein levels can reflect disturbances in cellular homeostasis and physiological functions [56]. Therefore, the observation of unchanged protein levels suggests that the exposure to ALPI, PU, and their combination did not result in significant disruptions to these essential cellular processes at the level of protein synthesis or degradation in planarians.

Additional limitations to the present research exist in a sense of potential lack of power to detect smaller effect sizes or the implications of using ANOVA when multiple comparisons are made. However, while the sample size was enough to observe statistical differences in regard to locomotor velocity, lack of statistical differences could be observed for other analysed parameters, possibly due to the high variability of the data and low number of replicates/sample size. Nevertheless, the analysis of all biomarkers showed that the activities of ETS, ChE, GST, and CAT, as well as the levels of TG and protein (Table 3 and Table 4) exhibited a high range of variation on planarians exposed to ALPI, PU, and ALPI + PU. Moreover, the weight and length of planarians (Table 2) and the biomarker of oxidative damage LPO were not significantly changed. Therefore, the impairment of the locomotor activity of ALPI + PU might be not only associated with previously discussed neurotoxic potential of the pollutants, but also to the energetic costs associated with non-significant alterations that are highlighted by the range response of organisms concerning energy consumption, detoxification, and antioxidant capacity. In view of environmental implications, decreased locomotor activity in planarians due to toxic exposure can have cascading effects on population dynamics by altering reproductive behaviours; reduced movement can limit access to food sources or suitable habitats; slower or less active planarians may be outcompeted by more mobile organisms for resources. Furthermore, decreased locomotor activity could make planarians more vulnerable to predation, reducing their survival rates and potentially altering predator–prey dynamics and thus the overall ecosystem structure and function. It is plausible to consider that the impairment of locomotor activity observed in response to ALPI + PU exposure may involve a combination of direct neurotoxic effects of the pollutants on the nervous system and the indirect energetic costs associated with the organisms’ adaptive responses to mitigate toxic effects. Thus, locomotor activity seems to be an integrated and cumulative biomarker of several changes in the cell physiology response to relatively low concentrations of both pollutants.

## 4. Conclusions

In conclusion, our research involving the exposure of *G. tigrina* to ALPI and PU revealed a notable decrease in locomotor activity while other molecular biomarkers (oxidative stress biomarkers and metabolic response) remained unaffected. This observation highlights the importance of including behavioural indicators in toxicological assays when assessing the effects of pollutants. Behavioural changes can serve as sensitive early indicators of environmental stressors, offering integrative and cumulative valuable insights into organism responses that may not be captured by molecular biomarkers alone. Furthermore, incorporating behavioural endpoints into toxicological assessments allows for a more comprehensive understanding of the overall impact of pollutants on organisms. By examining behavioural responses alongside molecular biomarkers, researchers can better elucidate potential synergistic or antagonistic effects of organic pollutants. These effects may manifest differently at the behavioural level compared to molecular endpoints, highlighting the necessity of a multidimensional approach in assessing ecological risks. Moreover, our study highlights the need to study in depth the single and combined ecophysiological effects of PU and ALPI using several concentrations and exposure periods. Further investigations could involve exploring different concentrations, exposure periods, and/or assessing recovery post exposure to evaluate the potential reversibility of the observed effects, providing a more comprehensive understanding of the impact of toxic substances on planarian physiology and behaviour.

## Figures and Tables

**Table 1:** Chemical parameters of commercial natural water in which planarians, *G. tigrina*, were kept during the exposure experiment under controlled conditions.

Parameter	Concentration (mg L^−1^)
Silicon dioxide, SiO_2_	16 ± 10
Hydrocarbonate ions, HCO_3_^−^	11.7 ± 0.60
Chloride ions, Cl^−^	2.9 ± 0.20
Sodium ions, Na^+^	5.2 ± 0.20
Calcium ions, Ca^2+^	1.3 ± 0.10

**Table 2:** Descriptive statistics on biomechanical responses—locomotor velocity (crossed gridlines min^−1^), body mass (g), and body length (mm) in planarians, *G. tigrina* exposed to 10 mg L^−1^ flame-retardant aluminium diethylphosphinate (ALPI), and 10 μg PU mg^−1^_liver_ plastic polymer polyurethane (PU), and the combination of ALPI and PU.

		Control	ALPI	PU	ALPI + PU
	n	10	10	10	10
Locomotor velocity	Min	4.00	3.00	6.00	3.00
(crossed gridlines min^−1^)	Max	15.00	15.00	15.00	10.00
	Range	11.00	12.00	9.00	7.00
Body mass (g)	Min	0.008	0.008	0.008	0.006
	Max	0.018	0.015	0.019	0.018
	Range	0.010	0.006	0.011	0.012
Body length (mm)	Min	10.00	11.00	10.00	11.00
	Max	15.00	15.00	13.00	14.00
	Range	5.00	4.00	3.00	3.00

ALPI—aluminium diethylphosphinate; PU—polyurethane; n—sample size; min—minimum value; max—maximum value.

**Table 3:** Descriptive statistics of oxidative stress biomarkers—glutathione S-transferase (GST, nmol min^−1^ planarian^−1^), catalase (CAT; µmol min^−1^ planarian^−1^), total glutathione (TG, nmol planarian^−1^), and lipid peroxidation (LPO, nmol planarian^−1^) in planarians, *G. tigrina* exposed to 10 mg L^−1^ flame-retardant aluminium diethylphosphinate (ALPI), 10 μg PU mg^−1^_liver_ plastic polymer polyurethane (PU), and the combination of ALPI and PU.

		Control	ALPI	PU	ALPI + PU
	n	10	10	10	10
GST (nmol min^−1^ planarian^−1^)	Min	6.87	0.96	3.92	5.49
	Max	31.24	34.54	36.48	25.69
	Range	24.37	33.58	32.56	20.20
CAT (µmol min^−1^ planarian^−1^)	Min	17.88	15.87	17.04	22.95
	Max	49.60	46.15	58.25	61.50
	Range	31.72	30.28	41.21	38.55
TG (nmol planarian^−1^)	Min	58.31	57.67	48.07	50.43
	Max	77.33	89.97	77.17	82.87
	Range	19.02	32.30	29.10	32.44
LPO (nmol planarian^−1^)	Min	0.79	0.70	0.52	1.11
	Max	10.62	8.89	7.43	10.22
	Range	9.82	8.19	6.91	9.11

ALPI—aluminium diethylphosphinate; PU—polyurethane; GST—glutathione S-transferase; CAT—catalase; TG—total glutathione; LPO—lipid peroxidation; n—sample size; min—minimum value; max—maximum value.

**Table 4:** Descriptive statistics of metabolic biomarker response—cholinesterase activity (ChE, nmol min^−1^ planarian^−1^), electron transport system (ETS, mJ h^−1^ planarian^−1^), lactate dehydrogenase activity (LDH, nmol min^−1^ planarian^−1^), and protein content (mg planarian^−1^) in planarians, *G. tigrina* exposed to 10 mg L^−1^ flame-retardant aluminium diethylphosphinate (ALPI), 10 μg PU mg^−1^_liver_ plastic polymer polyurethane (PU), and the combination of ALPI and PU.

		Control	ALPI	PU	ALPI + PU
	n	10	10	10	10
ChE (nmol min^−1^ planarian^−1^)	Min	32.89	31.21	15.38	27.39
	Max	65.62	123.20	94.79	87.74
	Range	32.73	92.01	79.41	60.35
ETS (mJ h^−1^ planarian^−1^)	Min	242.20	269.60	239.20	499.70
	Max	1473.00	1227.00	1068.00	1564.00
	Range	1231.00	957.30	829.20	1064.00
LDH (nmol min^−1^ planarian^−1^)	Min	7.33	8.98	6.76	8.26
	Max	42.39	45.90	48.39	33.47
	Range	35.06	36.92	41.64	25.21
Protein (mg planarian^−1^)	Min	0.51	0.51	0.44	0.58
	Max	1.37	1.43	2.13	1.50
	Range	0.86	0.92	1.69	0.92

ALPI—aluminium diethylphosphinate; PU—polyurethane; ChE—cholinesterase; ETS—electron transport system; LDH—lactate dehydrogenase; n—sample size; min—minimum value; max—maximum value.

## Data Availability

All data were included in the manuscript.
